# Mental health among female sex workers: A cross-sectional survey (study design)

**DOI:** 10.1192/j.eurpsy.2025.1484

**Published:** 2025-08-26

**Authors:** O. Kalinowski, A. Lotysh, G. Kaya, F. Kroehn-Liedtke, L. K. Zerbe, H. Mihaylova, W. Rössler, M. Schouler-Ocak

**Affiliations:** 1 Department of Psychiatry and Neurosciences, Psychiatric University Clinic of Charité at St. Hedwig Hospital, Berlin, Germany

## Abstract

**Introduction:**

“Sex work” refers to the provision of sexual services in exchange for monetary or material compensation. The exact number of sex workers in Germany, where prostitution is legal, remains uncertain, but estimates suggest it could be as high as 700,000. The majority of the group is female and has a migration background. Sex work covers different areas. These include work in brothels and strip clubs, street prostitution, hostess services and work in the porn industry. Previous research into sex work has mainly focused on sexually transmitted diseases. While results from Switzerland indicate elevated prevalences of depression, anxiety disorders, and substance use among sex workers, to date, there has been no research specifically addressing mental illnesses among sex workers in Germany.

**Objectives:**

The research project aims to fill this gap by determining the 1-year prevalence as well as risk and resilience factors for mental illness among sex workers in Berlin. The research findings aim to enhance psychosocial care services for sex workers and foster meaningful contributions to the broader social discourse.

**Methods:**

The socio-demographic composition of the sex worker population is constantly shifting, making it difficult to obtain a truly representative random sample. Quota sampling offers the closest alternative, with interviewees being approached at various workplaces. Additionally, we ensure a diverse age range is included in the study. A total of 500 participants will be surveyed using structured interviews. The interview consists of a sex work questionnaire, standardized research instruments, and a mental health screening. The sex work questionnaire gathers data on work type and intensity, socio-demographic characteristics, and individual access to counseling and support services. Standardized instruments include assessments for personality (TIPI), coping behaviors (Brief COPE), health-related quality of life (SF-12), stigmatization experiences (PASS 24), and childhood trauma (CTQ). The mental health screening will be conducted using the Mini-DIPS.

**Results:**

The submitted poster will offer a detailed overview of the study design and methodologies. In addition, preliminary results will be shared, shedding light on the mental health status and access to healthcare services among sex workers.

**Conclusions:**

This pioneering study seeks to address a significant gap in current research. The final findings will form the foundation for more in-depth analysis and guide future recommendations to improve support services for this population.

**Disclosure of Interest:**

None Declared

